# High-Dimensional Analysis of Acute Myeloid Leukemia Reveals Phenotypic Changes in Persistent Cells during Induction Therapy

**DOI:** 10.1371/journal.pone.0153207

**Published:** 2016-04-13

**Authors:** Paul Brent Ferrell, Kirsten Elizabeth Diggins, Hannah Grace Polikowsky, Sanjay Ram Mohan, Adam C. Seegmiller, Jonathan Michael Irish

**Affiliations:** 1 Department of Medicine, Division of Hematology/Oncology, Vanderbilt University Medical Center, Nashville, Tennessee, United States of America; 2 Department of Cancer Biology, Vanderbilt University, Nashville, Tennessee, United States of America; 3 Department of Pathology, Microbiology, and Immunology, Vanderbilt University Medical Center, Nashville, Tennessee, United States of America; University of Campinas, BRAZIL

## Abstract

The plasticity of AML drives poor clinical outcomes and confounds its longitudinal detection. However, the immediate impact of treatment on the leukemic and non-leukemic cells of the bone marrow and blood remains relatively understudied. Here, we conducted a pilot study of high dimensional longitudinal monitoring of immunophenotype in AML. To characterize changes in cell phenotype before, during, and immediately after induction treatment, we developed a 27-antibody panel for mass cytometry focused on surface diagnostic markers and applied it to 46 samples of blood or bone marrow tissue collected over time from 5 AML patients. Central goals were to determine whether changes in AML phenotype would be captured effectively by cytomic tools and to implement methods for describing the evolving phenotypes of AML cell subsets. Mass cytometry data were analyzed using established computational techniques. Within this pilot study, longitudinal immune monitoring with mass cytometry revealed fundamental changes in leukemia phenotypes that occurred over time during and after induction in the refractory disease setting. Persisting AML blasts became more phenotypically distinct from stem and progenitor cells due to expression of novel marker patterns that differed from pre-treatment AML cells and from all cell types observed in healthy bone marrow. This pilot study of single cell immune monitoring in AML represents a powerful tool for precision characterization and targeting of resistant disease.

## Introduction

Acute myeloid leukemia is one of the deadliest adult cancers. The five-year overall survival is 21.3% for all ages and 4.6% for individuals 65 and older [1]. Current standard of care therapy has remained relatively unchanged over the last 30 years despite efforts to improve these poor outcomes [2]. AML genetic heterogeneity has been well characterized as contributing to poor outcomes [3–5], and longitudinal genetic analyses have suggested multiple models of clonal evolution to explain disease aggressiveness [6, 7]. While it is clear that cell subsets within a pretreatment leukemia cell population have differential responses to therapy, it is not known to what extent genetic and non-genetic cellular features confer these differential responses. A single-cell understanding of AML therapy response over time during early treatment will characterize how different treatments reprogram AML cell phenotypes and impact clonal dynamics. Immediate post-treatment changes may have lasting impacts on long term outcomes, and a better understanding of how AML cells change following treatment may highlight key targets of opportunity for new treatments. Mass cytometry and unsupervised tools from machine learning provide a new opportunity to comprehensively characterize cellular heterogeneity and improve our understanding of how different treatments impact AML cell biology. In particular, it would be useful to characterize AML cells that remain immediately following treatment and determine whether they are distinct in a way that might be therapeutically targeted.

Immunophenotype characterization by flow cytometry has become part of standard of care in AML for diagnosis and disease monitoring, and standard antibody panels have been published for traditional fluorescence flow cytometry used in clinical pathology [8, 9]. A key strength of flow cytometry is the ability to measure several independent properties on each cell and to use complex combinations of these quantitative measurements to classify or isolate cells of interest [10, 11]. Surface antigens, such as CD34 and CD123, have been extensively studied individually or in small combinations, but reported associations with clinical outcome are numerous and often conflicting [12]. Additionally, leukemia stem cells (LSCs) and stem-ness properties likely play a significant role in therapy resistance and leukemia persistence in AML [13]. Furthermore, the markers that characterize an AML at diagnosis may shift during treatment and be changed dramatically in the case of minimal residual disease (MRD) or relapse [14]. Small antibody panels focused on positive identification of AML cells are susceptible to overlooking AML clones that undergo antigenic changes.

In contrast, measurement of 30 or more features by mass cytometry [15] can comprehensively characterize normal myeloid cell populations and, in combination with unsupervised machine learning tools, robustly characterizes all non-AML cells and distinguishes them from AML blasts [16]. Mass cytometry thus gives the potential for improved ability to define subsets throughout therapy. Because high-dimensional mass cytometry generates significantly more data than a traditional flow cytometry experiment, it therefore creates the need for new data processing and visualization tools. Computational tools, especially unsupervised algorithms, organize and display high dimensional data in a way not possible with traditional, supervised gating techniques [17–20]. One such algorithm, viSNE, has been shown to be robust in its ability to distinguish both healthy and leukemia subsets, showing great promise for research and clinical analysis and visualization of cytometry data [19, 21]. viSNE creates a phenotypic map of cells from an individual sample, or collection of samples, enabling visualization of phenotypic relationship between individual cells [19]. Furthermore, we have validated and published methods to use this tool to analyze both healthy and leukemia samples [22]. Given viSNE’s particular strength in visualization of high dimensional single cell data, it is well-suited to identify subtle or large changes in marker expression across several samples. Additionally, cells with unexpected phenotypes are routinely overlooked in manual analysis and viSNE captures many of these overlooked cells [19, 23].

We present a comprehensive single cell view of AML that tracks changes in the bone marrow and blood over time before, during, and after treatment and relates differences in cellular phenotype between patients over time and between cells from AML patients and healthy donors. From each patient, up to 12 samples of blood and marrow were obtained before, during, and after induction chemotherapy (n = 46 total samples; 14 marrow and 32 blood from 5 individual patients). This analysis represents a pilot of in-depth longitudinal monitoring of AML during induction therapy with mass cytometry. Additionally, machine learning analysis tools and mass cytometry were used to create a phenotypic stem-ness index for AML and applied to evaluate longitudinally collected patients samples.

## Materials & Methods

### Patients and Consent

Patients with suspected AML were consented for protocol sample collection. Healthy bone marrow was obtained from leftover sample from diagnostic analysis of non-cancerous tissue in the Vanderbilt Immunopathology core. All specimens were obtained in accordance with the Declaration of Helsinki following protocols approved by Vanderbilt University Medical Center (VUMC) Institutional Review Board (IRB). For each patient participating in the collection protocol, signed, written consent was obtained via a written consent form that was also approved by the VUMC IRB. Eligibility criteria included > = 18 years of age with suspected acute myeloid leukemia undergoing clinical evaluation at VUMC. Patients diagnosed with AML, excluding acute promyelocytic leukemia, who were treated with intensive induction chemotherapy, were eligible for complete collection protocol, regardless of induction regimen (individual regimens noted in Table A in S1 file). Basic clinical characteristics for each patient are listed in Table B in S1 file. One normal bone marrow was obtained from Vanderbilt Hematopathology Lab.

**Fig 1:**
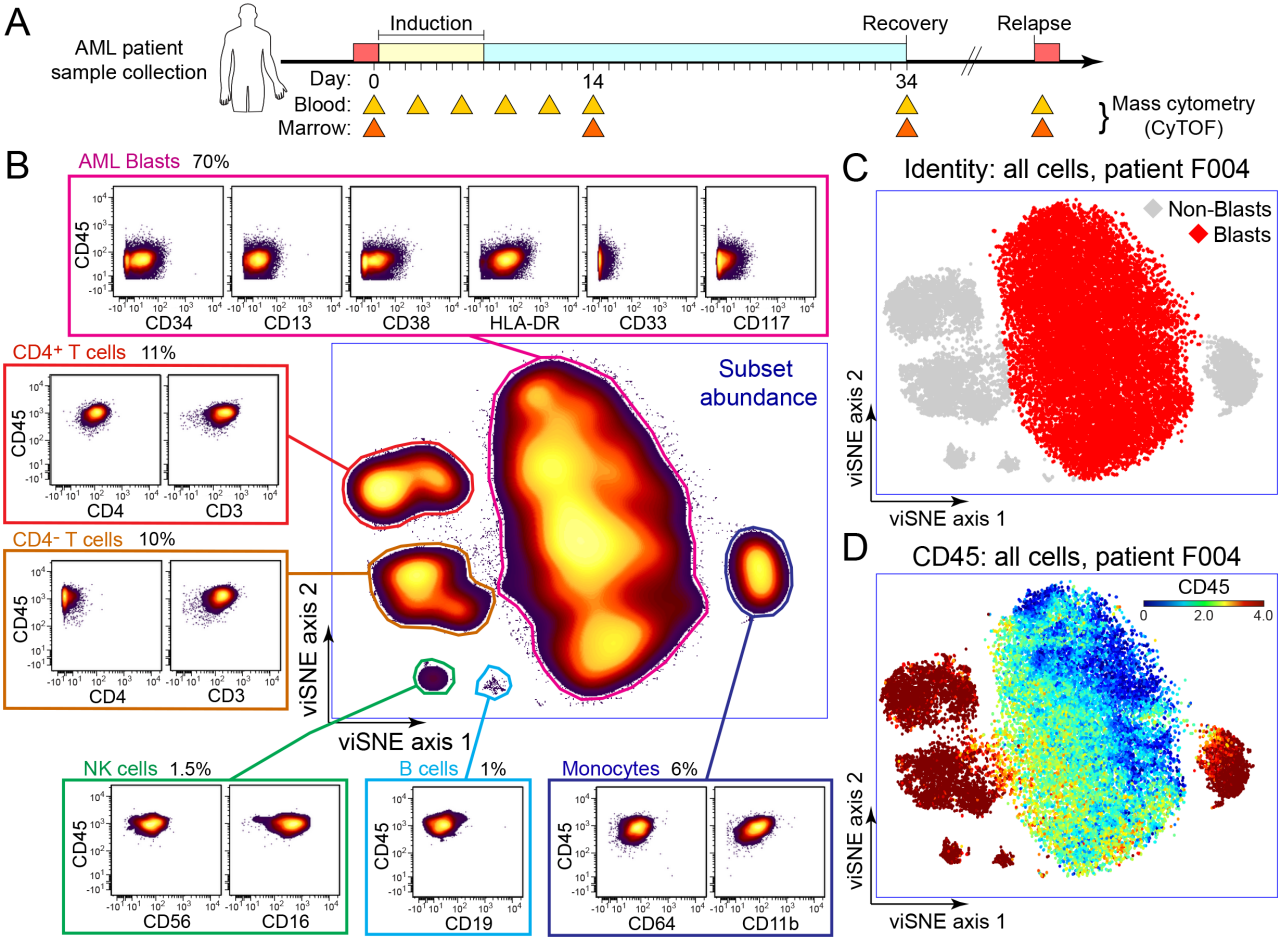
Overview of mass cytometry phenotyping in early AML therapy. (A) A timeline of AML induction shows blood and bone marrow collection goals for phenotypic comparisons as in Fig 2. Samples collected and analyzed for individual patients are listed in Fig 3 and Figure A in S1 File. (B) viSNE analysis of all live cells from the diagnosis marrow of one AML patient F004 is shown. Cells were arranged on the viSNE map along unitless x and y viSNE axes according to 27-dimensional phenotype (Table A in S1 File) so that phenotypically similar cells were placed near each other. Cellular abundance is indicated with a shaded contour plot where outliers start at 10% and each 2% contour is shaded a lighter color from purple to yellow. (C) On the same viSNE axes as in (B), diagnostic bone marrow cells from patient F004 were graphed and shaded according to identity determined by immunophenotype. AML blast cells were shaded red and non-blast cells were shaded grey. (D) On the same viSNE axes as in (B), diagnostic bone marrow cells from patient F004 were graphed and shaded according to expression of CD45 on a rainbow heatmap (log-like arcsinh_15_ scale).

After patients were consented, bone marrow and peripheral blood samples were obtained per protocol. Peripheral blood samples were collected pre-treatment at time of diagnosis or initial evaluation (Day 0), every 2–3 days during the first two weeks after the start of treatment, at day 14 (Day 14, also termed mid-induction; induction chemotherapy treatment ended for all patients before day 14), and at time of hematologic recovery, if applicable (Fig 1A). Bone marrow (BM) was collected at all clinically indicated time points, including pre-treatment Day 0, Day 14, and hematologic recovery, if applicable.

**Fig 2:**
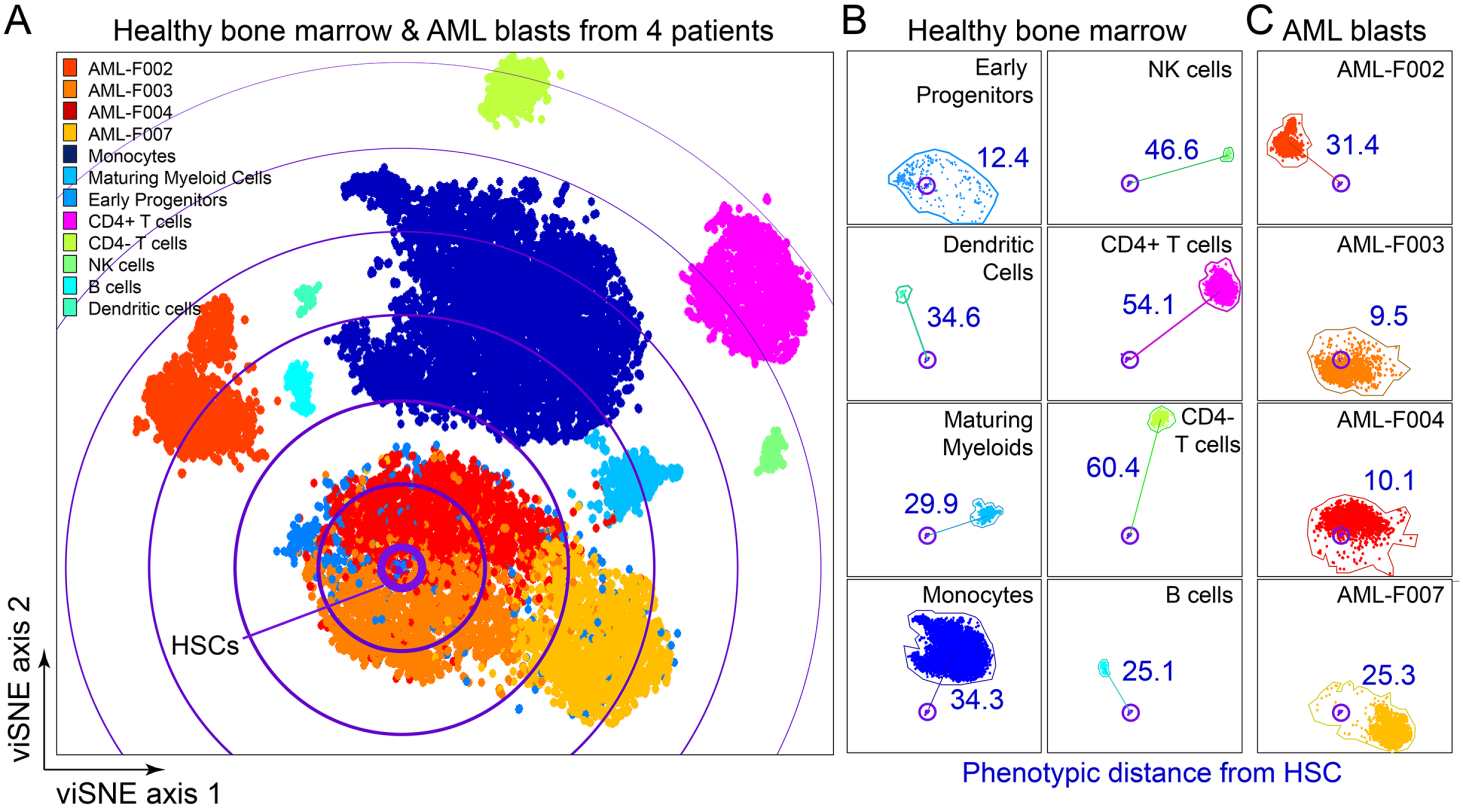
Phenotypic distance from hematopoietic stem cells distinguishes healthy cell populations and AML blasts from different individuals. (A) A 27-dimensional viSNE analysis compares an equivalent number of live cells from normal bone marrow and from each of four AML patient bone marrow samples obtained at diagnosis prior to treatment. (B) 27-dimensional phenotypic distance of normal bone marrow mononuclear populations from healthy hematopoietic stem cells (HSCs) was measured in the viSNE analysis from (A) and is shown in blue. (C) As in (B), the HSC distance for the blast populations from four AML patients was measured and is shown in blue.

**Fig 3:**
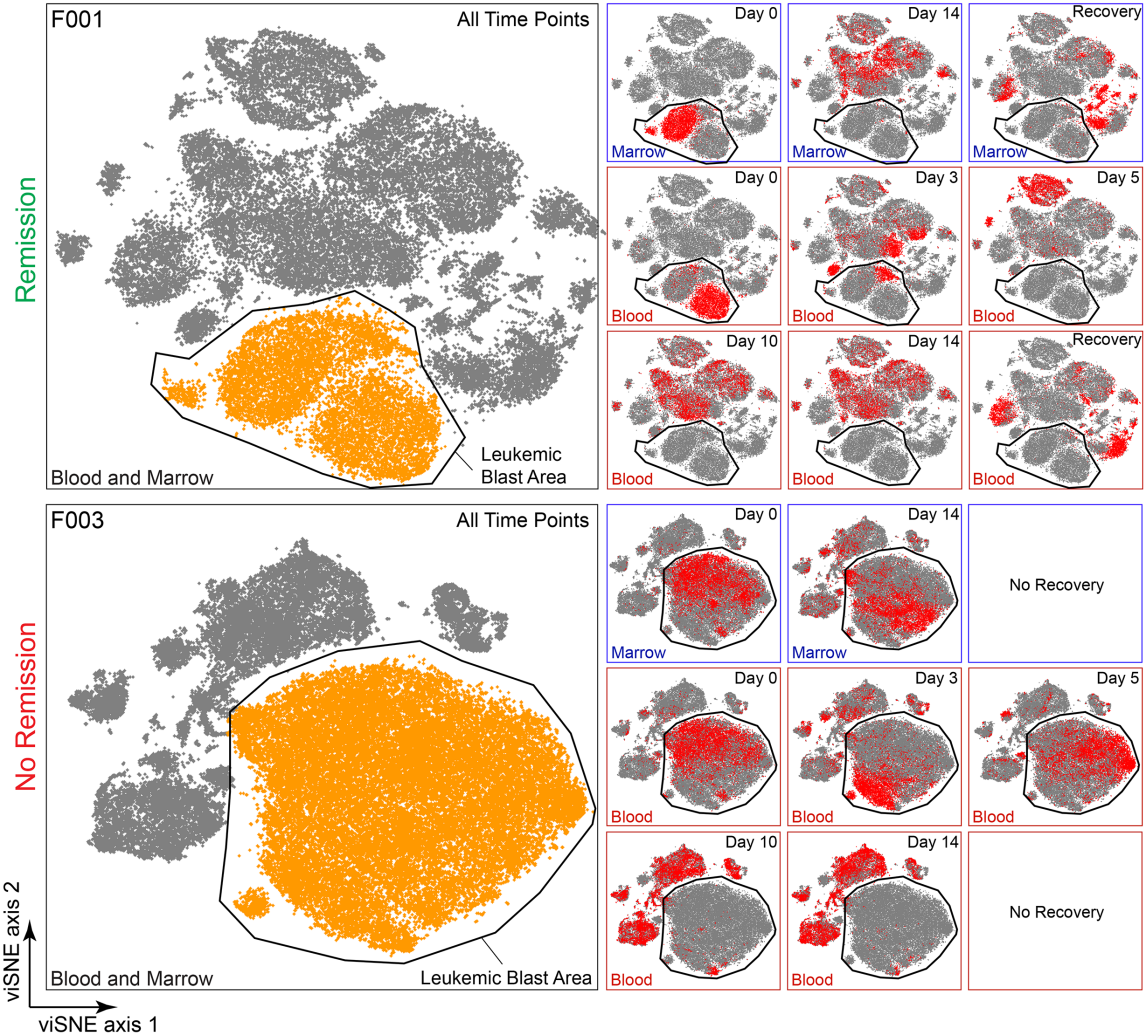
Computational analysis of samples throughout induction allows visualization of both remission and persistent AML. 27-dimensional viSNE analysis of all live cells from all sample collection time points for two AML patients is shown (left). Clinical response is indicated for each patient on the left. Leukemic blast areas were determined by location of cells at diagnosis and analysis of marker expression, as in Fig 1. At right, cells taken from the time point shown (red) were compared to all cells (grey). Differences in the location of cells within the viSNE map resulted from changes in protein expression.

### Sample Processing and Preservation

Once obtained, samples underwent immediate density gradient separation of mononuclear cells using BD Vacutainer^®^ CPT^™^ Cell Preparation Tube with Sodium Heparin (BD Biosciences, Franklin Lakes, NJ). The separated mononuclear cells were then pelleted with low speed centrifugation (200 x g) and aliquoted into multiple cryotubes in an 88% fetal bovine serum (FBS) + 12% DMSO solution. Samples were stored at -80 C for 24–72 hours prior to long-term storage in liquid nitrogen.

### Mass Cytometry

Aliquots of cryopreserved samples were thawed into 10ml of warm media (90% RPMI 1640 (Mediatech, Inc., Manassas, VA) + 10% fetal bovine serum (Gibco^®^ standard FBS, life technologies, Grand Island, NY)), pelleted by centrifugation at 200 x *g*, washed with warm media and pelleted again at 200 x *g* before resuspension in flow cytometry tubes with warm media (Falcon 2052, BD-Biosciences, San Jose, CA) and allowed to rest for 30 minutes in a 5% CO_2_ incubator at 37°C. Each rested sample was then pelleted at 200xg, washed with phosphate buffered saline (HyClone^®^, HyClone Laboratories, Logan, UT), pelleted and resuspended in cell staining media (CSM = PBS + 1% bovine serum albumin (BSA)) (Fisher Scientific, Fair Lawn, NJ). Cells were stained with a mass cytometry antibody panel of 27 antibodies (DVS Sciences, Sunnyvale, CA) designed based on inclusion of both consensus standard of care immunophenotyping panels for AML, as well as antibodies that allowed identification of non-AML PBMC (Table A in S1 file) [8]. A master mix of these antibodies was added to each sample to give a final staining volume of 50μL and incubated at room temperature for 30 minutes. Cells were then washed twice, first with CSM and then with PBS and then permeabilized in -20°C 100% methanol for 20 minutes. Following permeabilization, cells were washed, stained with 250 nM Iridium intercalator [24] (Fluidigm, San Francisco, CA) for 16 hours at 4°C, washed twice in PBS, and then re-suspended in 500 μL ddH_2_O for CyTOF analysis. Samples were analyzed using a CyTOF 1.0 at the Vanderbilt Flow Cytometry Shared Resource.

### Data Analysis

Mass cytometry data (.fcs) files were evaluated using R (version 2.5.2, The R Foundation for Statistical Computing), viSNE in MATLAB (version R2013b, The Mathworks, Inc.), and Cytobank software (Cytobank Inc.) [19, 25]. Files from each patient were mapped using the viSNE MATLAB graphical user interface, *cyt*, available at http://www.c2b2.columbia.edu/danapeerlab/html/cyt.html. Files used for viSNE co-analysis had the same number of cells randomly sampled from each file, unless very few cells were collected for a particular time point, in which case all cells were used in viSNE analysis.

### Measuring Hematopoietic Stem Cell Distance within viSNE

Within a given viSNE map, the hematopoietic stem cell distance (HSCD) for a given cell was calculated as the standard Euclidean distance formula $\left(\sqrt{{\left(x_{2}-x_{1}\right)}^{2}+{\left(y_{2}-y_{1}\right)}^{2}}\right)$ where *x*_1_, *y*_1_ was the coordinate location of a cell on the viSNE map’s t-SNE axes and *x*_2_, *y*_2_ was the center of the healthy HSC population on the same viSNE map. In particular, CD34^+^ CD38^lo/-^ hematopoietic stem cells were used as a common reference point for comparing non-leukemia subsets and phenotypically diverse AML samples. The center of the healthy HSC population was determined by averaging the x and y coordinates of the HSCs in the viSNE map. A population’s HSC distance was calculated as the mean HSC distance of all cells in that group. For comparison of healthy and leukemia inter-sample heterogeneity, mass cytometry .fcs files of healthy PBMC [26] and bone marrow [19] were used from published sources and remapped in separate viSNE analyses. Distances of common cell populations were measured from designated cells populations, HSC in the case of marrow samples and CD4+ T cells in the case of PBMC. Distances of these populations found in each sample were measured from reference population as described above and median and interquartile range (IQR) values were calculated to give a measure of inter-sample heterogeneity within healthy and AML samples.

## Results

### Machine Learning and Mass Cytometry Separated AML Blast Cells from Non-Malignant Cells

**Fig 4:**
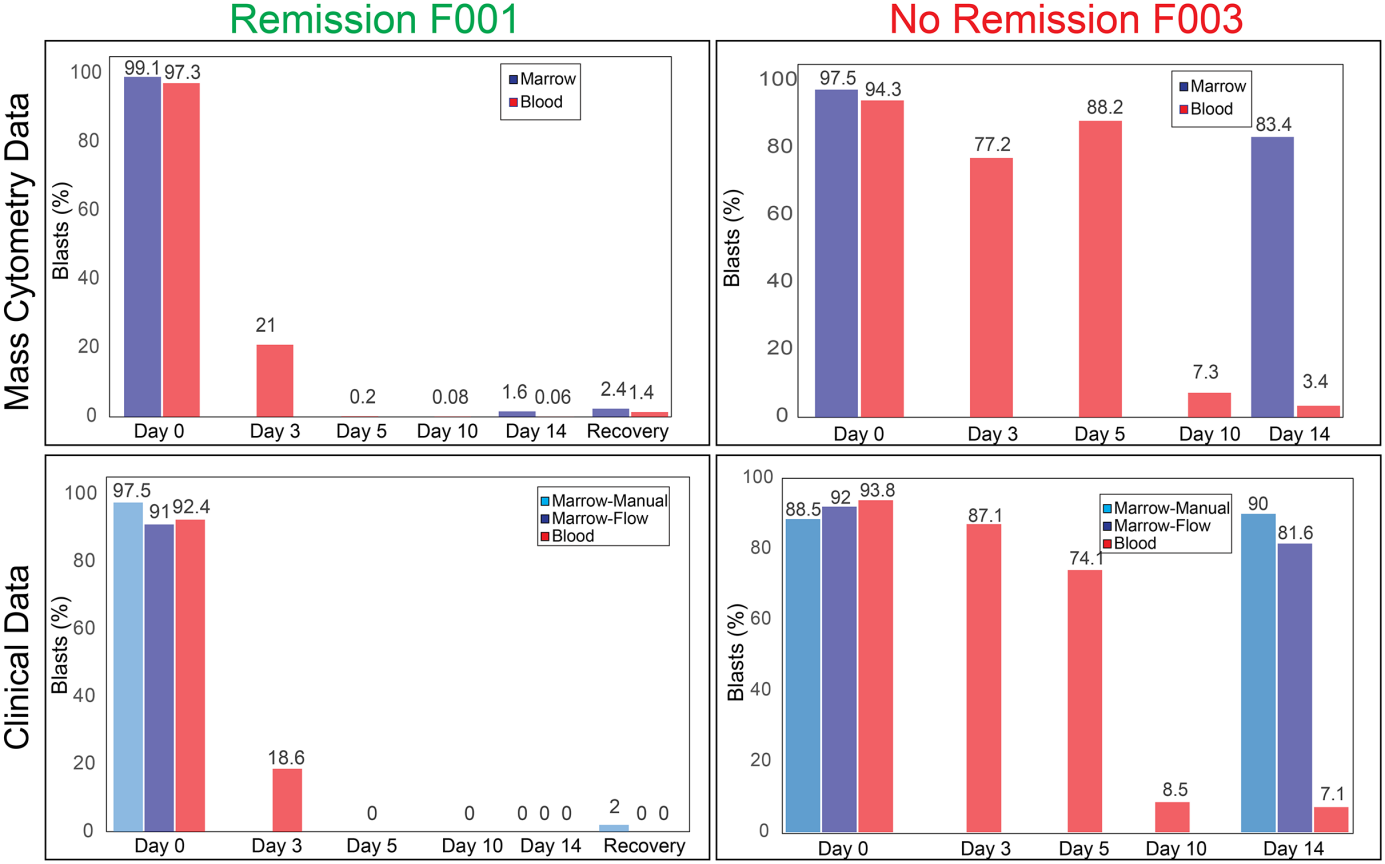
Analysis of immunophenotypic change throughout induction—F001 and F003. At top, mass cytometry quantification of blast percentage for each sample from two patients is shown. Below, clinical cytometry and microscopy data of each blood (clinical samples collected at different time on same day) and bone marrow (clinical and research samples collected at same time) sample throughout induction is shown. The patient whose samples are displayed on the left (F001) achieved remission (defined as <5% marrow blasts at recovery). On the right, a refractory patient’s samples are shown, in whom a very high blast percent were seen at Day 14.

AML blast cells were identified in viSNE using a combination of marker expression and localization on the 2–dimensional viSNE map. In each sample, a discrete group of cells was identified in the viSNE map characterized by a phenotype consistent with myeloid blasts (Fig 1B). Measured blast percentages were consistent with those reported for clinical pathology evaluation (data shown for F001 and F003 in Fig 4). Furthermore, other discrete islands within the map represented known populations of non-malignant mononuclear cells if present (Fig 1B and 1C). These islands were high for CD45 expression. Each island was characterized by analysis of each marker measured in that population and was assigned a cell identity (Fig 1B).

### viSNE Distance from Stem Cells Provides a Common Measure of Phenotypic Similarity to Stem Cells

In a viSNE map, proximity corresponds to phenotypic similarity according to the 27 measured proteins selected when creating the viSNE axes. To investigate the phenotypic similarity of non-leukemia mononuclear cells to stem cells, healthy bone marrow mononuclear cells and gated blast cells from four diagnostic AML bone marrow samples were mapped with viSNE (Fig 2A). Populations identified within a healthy sample included hematopoietic stem cells (HSCs), early progenitors (EPs), dendritic cells, maturing myeloid cells, monocytes, natural killer (NK) cells, CD4+ and CD4- T cells and B cells. Phenotypic distance from HSCs was then used to characterize healthy and AML population. Healthy NK cells and T cells were the most phenotypically distinct from HSC with distances of 46.6 (NK), 54.1 (CD4^+^ T cells), and 60.4 (CD4^-^ T cells). CD34^+^ CD38^+^ bone marrow cells comprised the majority of the EP population. EPs occupied the largest area of phenotypic space relative to the number of cells in the population, indicating that EPs were the most heterogeneous cell population observed. The mean distance of EPs from HSCs was 12.4, indicating that, on average, EPs were the healthy cell type most closely related to the HSCs, as expected. EPs were phenotypically similar to healthy stem cells when considering 27 protein features (Fig 2B). This calculated HSC distance was used to quantify the phenotypic distance of each patient’s AML blasts from known cell populations within healthy marrow.

### Diagnostic AML Blasts from Four Patients Differed in Phenotypic Distance from HSCs

Within the viSNE map, HSC distance was measured for AML blasts from four patients’ diagnostic samples (Fig 2C). AML blasts localized to one general area of the viSNE map; however, while samples from F003, F004, F007 were associated closely, AML blasts from patient F002 were phenotypically distinct and well separated from other AMLs (Fig 2A). In contrast with healthy early progenitor cells, AML blasts were more homogeneous in phenotype, as AML blasts occupied a smaller phenotypic area of the map than EPs (Fig 2A). This was despite the fact that AML blast populations contained approximately 5.4 times as many cells as the EP population in this analysis.

AML blasts from patients F003 and F004 were phenotypically similar to healthy HSCs and had very low distance values near 10. This result contrasted with that from AML patients F002 and F007, whose diagnostic blasts were farther from healthy stem cells and had distance values near 30 (Fig 2), highlighting the patient-to-patient diversity that can be detected with even a small cohort of patients. By comparing distance to HSC and other populations on the viSNE map, each patient’s AML blasts were characterized as phenotypically more similar to HSCs (F003, F004) or more distant from HSCs (F002 and F007) (Fig 2B).

To assess donor variation for this type of comparison for other cell subsets, distances on viSNE axes were compared for 5 sets of test and reference populations from this manuscript and others and reported as the interquartile range (IQR) and median across individuals. Little heterogeneity in viSNE distances was observed when comparing CD4+ T cells from healthy PBMC to B cells from healthy PBMC (IQR: 1.3, median: 38.1, N = 7). Similarly, relatively little heterogeneity was seen in comparisons of healthy BM B cells to healthy BM HSCs (IQR: 4.5, median: 10.1, N = 3), non-leukemia CD4+ T cells from AML BM to healthy BM HSCs (IQR: 2.7, median: 9.5, N = 3), and non-leukemia CD4+ T cells from AML BM to healthy BM HSCs (IQR: 2.4, median: 7.3, N = 3). All of these low interquartile ranges contrasted with the significantly greater heterogeneity seen when comparing AML blasts compared to healthy BM HSCs here (IQR: 18.6, median: 17.7, N = 4). Thus, the heterogeneity in viSNE distance was specific to the leukemic blasts and not observed in comparisons including non-leukemia cells from the same sample, healthy blood, or healthy bone marrow.

### Mapping Early Treatment Samples in viSNE Allowed Accurate Assessment of Blast Clearance

In order to assess whether this technique properly characterized blast clearance, samples over time from individual patients were mapped in patient-specific viSNE analyses. A leukemic blast area or island was identified in the viSNE map and treatment response was observed as the regression or persistence of cells within this area over time. In patients who achieved remission (based on clinical pathology assessment), >95% of cells in the recovery samples were outside of the leukemic blast area (Figs 3 and 4 and Figure A in S1 File).

Abundance and phenotype of AML cells changed significantly in the blood during the first days of treatment, with significant variability among patients (Fig 3 & Figure A in S1 File). Absolute numbers of all cell types, malignant and non-malignant, declined predictably in the blood of all patients by Day 14. Lymphocytes, predominantly T cells, were the most abundant cell population in all patients at Day 14, when leukocyte counts in marrow and blood are typically at their nadir. Leukemic blasts in the blood and marrow formed distinct islands in viSNE and these islands could be followed over time for each patient (“Leukemic Blast Area”, Fig 3 and Figure A in S1 File). Cells were observed largely to regress from this area in blood by Day 14 in all patients (>90%). The patient with the highest Day 14 peripheral blood blast proportion (F003) also had the highest percentage of blasts seen in the bone marrow (see below). Overall, the mass cytometry results were comparable to those obtained by clinical pathology analysis (Fig 4).

Day 14 bone marrow aspirate was obtained from patients when sufficient sample was available (patient F002 had inaspirable marrow at Day 14, though a core biopsy revealed no residual AML). Bone marrow from patients F003 and F004 was significantly involved with AML post-induction, and both patients required reinduction. Patient F003 never achieved remission despite two inductions and the initial Day 14 marrow was highly involved by AML (90% blasts by microscopy, 81% by clinical flow cytometry). This clinical finding was corroborated by mass cytometry and viSNE analysis, as both patients had cells mapped within the leukemic blast area at Day 14 (Fig 3 and Figure A in S1 File). For patient F004, residual cells occupied a tight area on the map and were phenotypically distinct from the majority of the AML cells in the diagnostic sample, both in the marrow and the blood (Figure A in S1 File). This residual population of AML cells did not persist at Day 14 of the second induction (Day 14–2) or at recovery biopsy after second induction, when the patient was found to be in remission. Thus, a population of leukemia cells for F003 and F004 persisted at the first bone marrow biopsy after induction chemotherapy (Day 14) and a change in overall AML cell phenotype was observed in this persistent population in both cases.

### Mass Cytometry Identified Phenotypically Distinct Persisting AML Blasts That Were Rare Pre-Treatment

**Fig 5:**
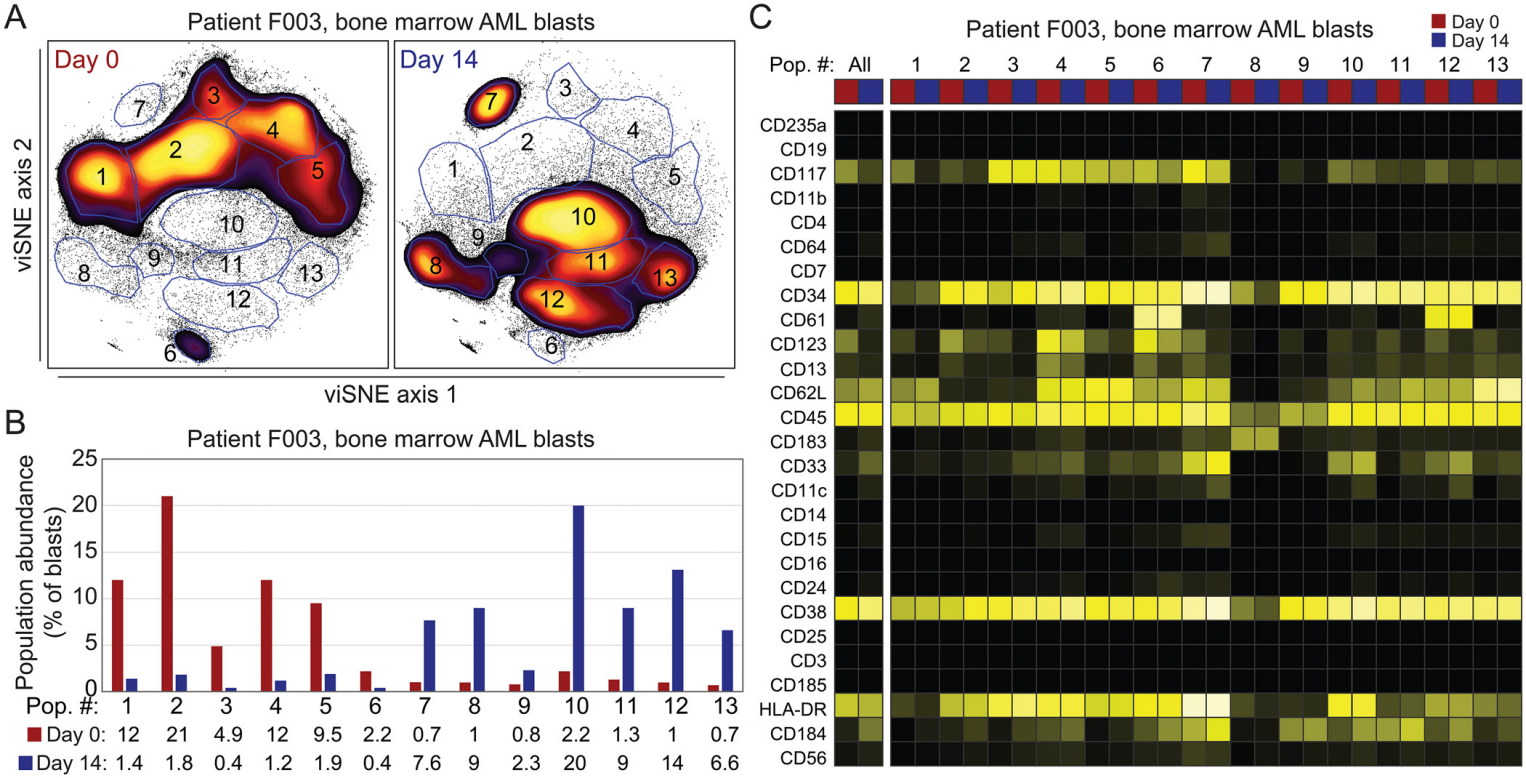
Rare subsets at diagnosis become prominent in persistent leukemia in a patient with refractory AML. (A) Blasts from Day 0 and Day 14 were gated out from prior viSNE maps for patient F003 (shown in Fig 3) and remapped in viSNE together. Gates were drawn around subpopulations and based on relative cell abundance, as in Fig 1B. (B) Percent of total blasts for each gate at both time points. (C) A heat map of median marker expression for each gate at both time points is shown.

In Day 14 bone marrow from patient F003, discrete populations of persisting cells made up the majority of the sample (80%). In order to perform a more direct comparison of pre-treatment (Day 0) and Day 14 AML blasts, these blast populations were gated from Day 0 and Day 14 bone marrow samples and remapped in viSNE. They were then visualized in order to demonstrate subpopulations based on cellular abundance (Fig 5A). Thirteen AML cell populations were identified based on phenotype and abundance in pre-treatment and post-treatment samples. Of these AML cell populations from patient F003, 6 populations were abundant pre-treatment and 7 totally distinct populations were abundant at Day 14 (Fig 5A).

Overall, the AML phenotype shifted markedly at Day 14. Over 85% of cells at Day 14 were grouped into 7 phenotypic regions as mentioned above. By contrast, fewer than 10% of the cells fell into these regions pre-treatment (Fig 5B). The subpopulations observed at Day 14 ranged in abundance from 2.3% to 20% of the AML cells. At diagnosis, in the pre-treatment sample, these populations constituted as little as 0.6% to 1.8% of total blasts (Fig 5B).

### Increased Expression of CD34, CD38 and CD184 Characterized AML at Day 14 While Subpopulations Displayed Significant Heterogeneity

**Fig 6:**
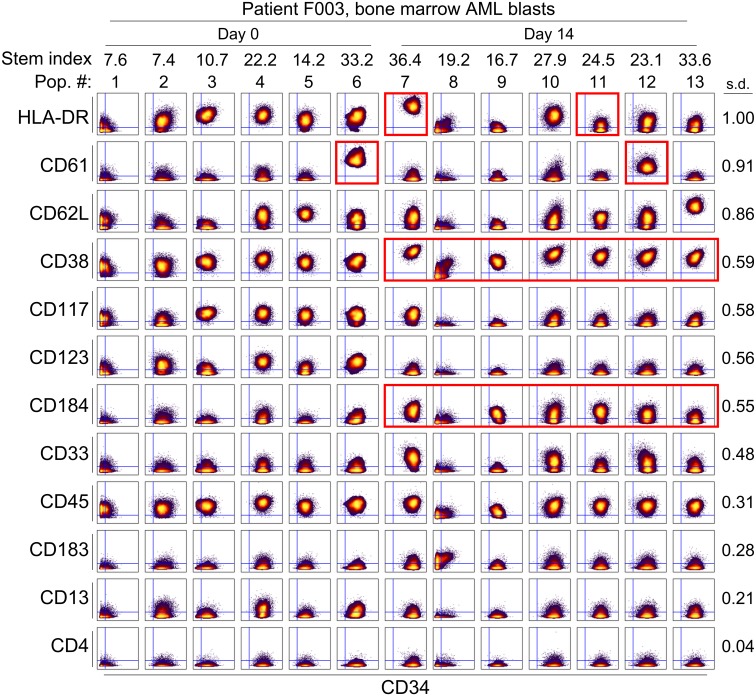
Single cell analysis of immunophenotype in AML subpopulations at diagnosis and mid-induction persistence. Biaxial density plots show the markers with the highest standard deviation across subsets compared with CD34 on individual cells in live bone marrow AML blasts from patient F003. Columns show AML blast populations identified (Fig 5) from Day 0 or Day 14, whichever was more abundant. Red boxes highlight key changes in protein expression discussed in the text. Standard deviation (SD) of each row’s marker across all populations is indicated to the right of the plots. Stem index (Fig 2) of each population is indicated above each column. CD34 and the top 11 most variable markers were graphed. CD4 was included as an example of a low expression marker that did not change.

Individual marker changes contributing to overall shift were then characterized. Several markers of leukemia stem cells were identified as contributing to the overall shift in phenotype in samples from patient F003 (Figs 5C and 6). Median marker expression within the 13 identified subsets was calculated and visualized as a heat map (Fig 5C). Prominent changes seen in AML cells persistent at Day 14 after the start of treatment included both overall increases and decreases in specific markers. Among these markers were CD34, CD38, and CXCR4/CD184, which respectively increased by 0.7, 0.9, and 0.6 fold on the log-like asinh_15_ scale. Expression of CD117 and CD123, markers associated with LSC ability, decreased in the persistent leukemia. In non-leukemic cells, expression of CD34, CD38, and major cell type identity markers (e.g. HLA-DR, CD4, CD19) did not significantly change over time on non-AML cells (all <0.2 fold, Figures B and C in S1 File).

Analyses limited to overall changes in the entire population of persistent cells belie the underlying heterogeneity seen in individual subpopulations of cells, where changes are quite diverse. Though CD117/*c-kit* expression showed a large decrease from initial Day 0 pre-treatment sample to Day 14 sample, the heat map and biaxial plots demonstrate that expression of this marker is heterogeneous and remains high in one subpopulation (population 7 in Figs 5C and 6). Subpopulation 7 is also notable for increased expression of CD33 and CD184/CXCR4, which are both quite low in all of the prominent pre-treatment populations (Fig 6). Based on this, it is apparent that a heterogeneous group of subpopulations constitute AML at diagnosis and in the early pre-treatment period. Thus, the overall shift in AML phenotype was driven by the differential abundance of phenotypically distinct subpopulations that were rare pre-treatment (Fig 5A–5C).

### Marker Expression Varied Among Subpopulations

To quantify variability within subpopulations, we calculated the standard deviation of each marker among all 13 subpopulations identified in samples obtained at Day 0 and Day 14. The most variable markers among the subpopulations are shown in Fig 6, with the exception of CD4, which was a negative, non-variable marker on these leukemia subpopulations. HLA-DR displayed the most variability (SD = 1.0), followed closely by CD61 (SD = 0.91) and CD62L (SD = 0.86). Expression of these markers ranged from high expression to little or no expression across the 13 populations, with variability seen in both diagnostic and Day 14 samples. Interestingly, population 8 was identified as having little or no expression of most markers that identified leukemic blasts, though this population was seen in both pre-and post-treatment samples and its constituent cells were identified in the initial gating schemes (Ir+, single cells).

### Persisting AML Cells Become Less Phenotypically Stem-Like

**Fig 7:**
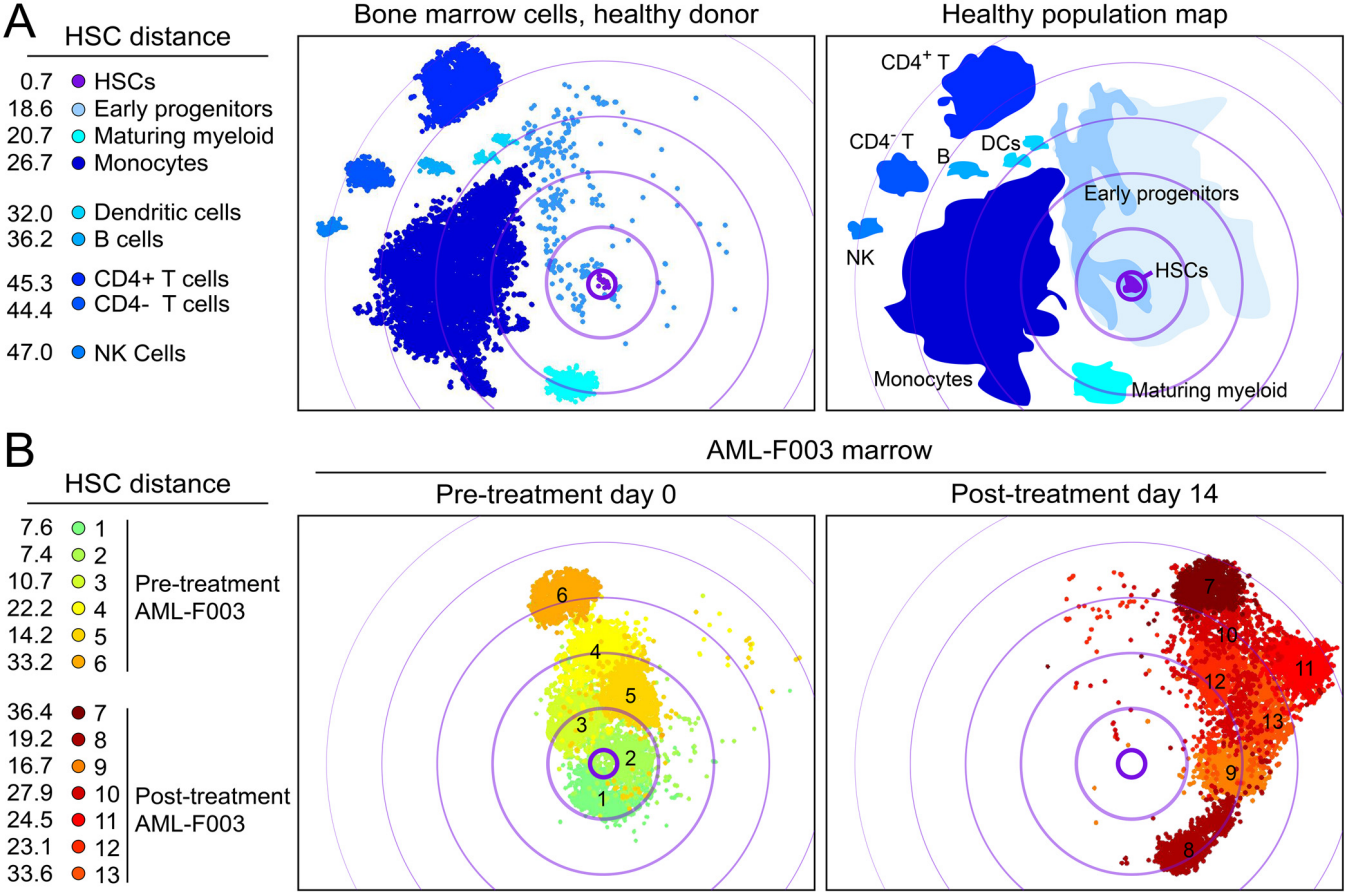
Blast populations become increasingly phenotypically different from both differentiated cells and non-malignant hematopoietic stem cells. 27-dimensional viSNE analysis compared normal bone marrow cells, AML blasts from Day 0 from patient F003 (subpopulations 1–6 only), and AML blasts from Day 14 from patient F003 (subpopulation 7–13 only). (A) Normal bone marrow mononuclear cells are shown (left). A cartoon outline of the major healthy subpopulations was identified and HSC distance measured (right). (B) Pre-treatment (Day 0) and post-treatment (Day 14) samples are compared same map as in (A) and HSC distance of each subpopulation is shown.

In order to understand how individual marker changes affected overall phenotypic diversity and identity with respect to known, healthy populations, leukemia blasts from Day 0 and Day 14 were mapped in viSNE with the normal healthy marrow population. Based upon the resultant viSNE map, phenotypic shifts were quantified and persistent AML blasts were shown to be less phenotypically similar to healthy HSCs (Fig 7B). For this analysis, predominant bone marrow blast populations at diagnosis (AML subsets 1–6) were mapped with normal marrow and major persisting populations from Day 14 (subsets 7–13) (Fig 7A–7B). At Day 14, the mean change in HSC distance was +10.0, indicating a phenotypic shift away from HSCs (Fig 7B). Taken together, these data indicate that the overall trend is for AML blasts to gain a novel phenotype that is distinct from those of healthy stem and progenitor cells in the bone marrow.

## Discussion

This work characterizes changes in AML blasts and healthy cells over time following induction chemotherapy according to 27 protein features, including the vast majority of diagnostic markers for this disease. A key benefit of this approach is that it reveals whether AML cells are cleared, phenotypically altered, or left unchanged after treatment. This approach directly measures the impact of treatment on the cellular milieu of the patient’s marrow and blood. By identifying persistent AML cells post therapy, mass cytometry and computational analysis provided a clear picture of AML subpopulation dynamics in the early therapy response and revealed the unexpected finding that AML “persister” cells can become significantly less phenotypically stem-like immediately following treatment. These results provide a foundational reference for understanding the reservoir of AML cells that evade treatment and provide a proof of concept for high dimensional single cell characterization of clinically relevant cell-surface molecules in AML.

While this study assessed samples from 5 patients, there are 46 total samples from these 5 patients that have been analyzed. Regardless, this pilot study assessed the feasibility of greatly expanded longitudinal monitoring of AML to capture differentiation and cell subset dynamics. AML displays significant phenotypic plasticity in the clinical setting [14]. However, this phenotypic heterogeneity is not well represented in focused fluorescent cytometry panels measuring between six and eight features. Here we do not intend to duplicate work done by large clinical flow consortia, such as EuroFlow, rather we intend to demonstrate the utility and feasibility of this approach [9, 27]. Larger studies of high-dimensional immune cell monitoring in AML are now needed to further validate this approach and to begin to understand treatment specific changes. This study demonstrates potential drawbacks of small fluorescence cytometry panels used to distinguish the leukemia blasts at during therapy—namely that changes in certain markers may be missed by small panels. The results of this study also indicate that immunophenotype in AML is highly plastic and can fundamentally shift in a matter of days in response to treatment to adopt a pattern that is both abnormal and not HSC-like. Furthermore, while this study was not designed to determine whether or not chemotherapy is altering cell phenotypic expression or selecting for persistent cells, the kinetics of early marker change in the peripheral blood indicate that phenotypic plasticity is possible.

This focused pilot study indicates that mass cytometry is feasible for clinical immune monitoring of treatment impact in blood cancers, such as AML. Furthermore, these findings are immediately translatable to longitudinal studies of other hematologic malignancies, chronic viral infections, and autoimmune disorders. Early phenotypic changes may also be evaluated in the peripheral blood blasts, when present, and could give key insight to early treatment success. It has been shown that early blasts decline in peripheral blood can predict complete remission rates in AML [28–30]. With added information of high-dimensional directionality and phenotypic distance changes, peripheral blood changes can be better characterized early and could become even more informative. However, because peripheral blasts can have a slightly different immunophenotype than bone marrow blasts (as in F001), changes in blasts post-therapy are likely best evaluated within the same tissue compartment. Visualization of high-dimensional relationships within viSNE allow for assessment of changes over time as well as noting any differences between different compartments (e.g., marrow vs. blood). The approach presented here also provides a new clinical tool for tracking and dissecting cellular identities and functional responses in clinical studies and translational research. For example, the markers identified here can now be used to sort persister-like cells from pre-treatment AML and determine whether these cells are genetically related to the persister cells. Recently published data reveals the decoupling of immunophenotype and signaling in AML samples at diagnosis and shows that the presence of “primitive” signaling profiles found in healthy HSCs likely more prognostic than phenotype [31]. Applying this finding to our data, we would predict that refractory AML adopts a more “primitive” signaling pattern post-treatment, regardless of phenotypic changes. In this way, application of signaling measurements to longitudinal monitoring in AML has great potential to reveal signaling and phenotypic markers of resistance that could improve disease targeting.

This study describes a novel phenotypic stem-ness index based on the high dimensional immunophenotype of normal and leukemia samples. It is possible that the majority of persistent leukemia cells are leukemia stem cells and their phenotypic changes reveal a level of plasticity in stem cell phenotype that cannot to be assessed outside of real world treatment situations. Importantly, some of this plasticity has been seen in vitro as well [13, 32]. It is possible that regardless of their “stem-ness”, as defined either by phenotype or function, these therapy resistant cells are the cells that warrant further investigation and therapeutic focus in the context of AML, as it is their persistence post-treatment that results in the ultimate death of patients. We have shown that subsets present in a refractory AML display increased expression of CD34, CD38, and CD184 while viSNE analysis showed that these cells “moved away” phenotypically from healthy stem cells and the bulk of the original disease. The sample from refractory patient F003 was from an early post-chemotherapy time point (Day 14 bone marrow) and it is not known if the leukemia cells continued changed in phenotype at later time points after therapy. While the AML cells clearly moved in a new immunophenotypic direction and adopted new features, they also maintained characteristics of the pre-treatment AML blasts. This could indicate some subtle maturation, however abnormal, was induced by the treatment within the blast population. LSCs have been shown to change immunophenotype. Although LSCs were initially described as Lin^-^ CD34^+^ CD38^lo/^, it is well-established that cells with leukemia initiating ability and stem-ness properties exist outside of the Lin^-^ CD34^+^ CD38^lo/-^ population [32–35]. This may be especially true in cases of refractory AML. Additionally, expression of CD184 has been linked to worse outcome in AML, and therapeutic targeting of CD184 has been reported [36, 37].

This refractory AML presented here demonstrated a large burden of persistent leukemia cells after therapy. These persister cells are common in AML, as evidence by MRD in patients who achieve a remission or as overt refractory disease. At no point did we see AML “collapse” into a phenotypically more similar (less heterogeneous) population or adopt a uniform stem or progenitor cell phenotype that could imply a bottleneck selection event. The response to treatment was typically an increase in diversity of phenotypes and movement away from traditional stem cell phenotypes. It may be that there is an inducible mechanism of phenotypic shift in AML cells caused by chemotherapy. This mechanism could depend on non-cell autonomous changes and inter-clonal interaction, as has recently been described in cancer cells [38]. This would contrast with the classic “selecting for a subclone” model and aligns with some recent observations based on gene sequencing, which highlight genetic and functional heterogeneity within AML at diagnosis and relapse [6, 7]. Further clarification of the functional differences in immunophenotypically distinct subsets of AML, particularly of intracellular signaling networks, is needed to identify novel therapeutic targets in this disease.

## Supporting Information

S1 File Supplemental Information. **Table of antibodies for mass cytometry**. Antibodies used for mass cytometry staining are listed in this table. Clone of the antibody along with designation of type and mass number are listed for each antibody. Antibody “type” in this table is based on markers recommendations for AML phenotyping by the Bethesda International Consensus Conference [39] (Table A). **Patient characteristics and clinical outcomes** (Table B). **Comprehensive analysis of AML therapy response kinetics**. viSNE analysis characterizes changes in leukemia cell phenotype over time for cells from all treatment times for three individuals. All cells from all clinical timepoints were analyzed using viSNE according to the 27 markers measured (Table A in S1 file). An AML blast area was identified as in Fig 1. Color indicates clinical time point and source, either bone marrow or blood. Patients F002 and F007 had very low blast percentages in the peripheral blood at diagnosis (Figure A). **Changes in individual markers over time during treatment on AML blasts from patient F003**. Biaxial plots summarize six clinical timepoints (rows) for 24 markers (sets) for the AML blast cells from patient F003, gated as shown in Fig 3. The indicated marker is plotted on the x-axis using the same arcsinh_15_ scale as in other figures (e.g. Fig 1B). Plot labels are omitted to save space. The y-axis is mass cytometry event length, which is used here to spread the events out in the y-axis to create a compressed band plot view that allows rare subsets to be observed (see e.g. CD235a) that would be obscured in a traditional 1D histogram view (Figure B). **Changes in individual markers over time during treatment on non-leukemia cells from patient F003**. As in Figure B in S1 File, biaxial plots summarize six clinical timepoints (rows) for 24 markers (sets) for the non-leukemia cells from patient F003, gated as everything not in the leukemia blast gate shown in Fig 3. The indicated marker is plotted on the x-axis using the same arcsinh_15_ scale as in other figures (e.g. Fig 1B). Plot labels are omitted to save space. The y-axis is mass cytometry event length, which is used here to spread the events out in the y-axis to create a compressed band plot view that allows rare subsets to be observed (see e.g. CD16) that would be obscured in a traditional 1D histogram view (Figure C).
(DOCX)
