# Supplementary material for: High-Dimensional Analysis of Acute Myeloid Leukemia Reveals Phenotypic Changes in Persistent Cells during Induction Therapy
Source: PLoS One. 2016 Apr 13;11(4):e0153207. doi: 10.1371/journal.pone.0153207 (PMC4830605; doi:10.1371/journal.pone.0153207)
Supplement: S1 File — Table of antibodies for mass cytometry. Antibodies used for mass cytometry staining are listed in this table. Clone of the antibody along with designation of type and mass number are listed for each antibody. Antibody “type” in this table is based on markers recommendations for AML phenotyping by the Bethesda International Consensus Conference [39] (Table A). Patient characteristics and clinical outcomes (Table B). Comprehensive analysis of AML therapy response kinetics. viSNE analysis characterizes changes in leukemia cell phenotype over time for cells from all treatment times for three individuals. All cells from all clinical timepoints were analyzed using viSNE according to the 27 markers measured (Table A in S1 file). An AML blast area was identified as in Fig 1. Color indicates clinical time point and source, either bone marrow or blood. Patients F002 and F007 had very low blast percentages in the peripheral blood at diagnosis (Figure A). Changes in individual markers over time during treatment on AML blasts from patient F003. Biaxial plots summarize six clinical timepoints (rows) for 24 markers (sets) for the AML blast cells from patient F003, gated as shown in Fig 3. The indicated marker is plotted on the x-axis using the same arcsinh15 scale as in other figures (e.g. Fig 1B). Plot labels are omitted to save space. The y-axis is mass cytometry event length, which is used here to spread the events out in the y-axis to create a compressed band plot view that allows rare subsets to be observed (see e.g. CD235a) that would be obscured in a traditional 1D histogram view (Figure B). Changes in individual markers over time during treatment on non-leukemia cells from patient F003. As in Figure B in S1 File, biaxial plots summarize six clinical timepoints (rows) for 24 markers (sets) for the non-leukemia cells from patient F003, gated as everything not in the leukemia blast gate shown in Fig 3. The indicated marker is plotted on the x-axis using the same arcsinh15 sca [file pone.0153207.s001.docx]

**Ferrell et al. – Supplementary Information S1 File**

| **Supplemental Table A –** AML mass cytometry panel | | |
| --- | --- | --- |
| **Target (Clone)** | **Type** | **Mass** |
| CD235a (HIR2) | β | 141 |
| CD19 (HIB19) | γ | 142 |
| CD117 (104D2) | α | 143 |
| CD11b (ICRF44) | α | 144 |
| CD4 (RPAT4) | β | 145 |
| CD64 (10.1) | β | 146 |
| CD7 (CD7-6B7) | α | 147 |
| CD34 (581) | α | 148 |
| CD61 (VI-PL2) | β | 150 |
| CD123 (6H6) | β | 151 |
| CD13 (WM15) | α | 152 |
| CD62L (DREG-56) | γ | 153 |
| CD45 (HI30) | α | 154 |
| CD183 (G025H7) | γ | 156 |
| CD33 (WM53) | α | 158 |
| CD11c (Bu15) | γ | 159 |
| CD14 (M5E2) | α | 160 |
| CD15 (W6D3) | α | 164 |
| CD16 (3G8) | α | 165 |
| CD24 (ML5) | γ | 166 |
| CD38 (HIT2) | β | 167 |
| CD25 (2A3) | β | 169 |
| CD3 (UCHT1) | γ | 170 |
| CD185 (51505) | α | 171 |
| HLA-DR (L243) | α | 174 |
| CD184 (12G5) | γ | 175 |
| CD56 (CMSSB) | α | 176 |
| Iridium | § | 191 |
| Marker type key:  α – Consensus AML  β – Secondary AML  γ – Other  § - Mass cytometry | | |

**Supplemental Table A.** Antibodies used for mass cytometry staining are listed in this table. Clone of the antibody along with designation of type and mass number are listed for each antibody. Antibody “type” in this table is based on markers recommendations for AML phenotyping by the Bethesda International Consensus Conference [[1](#_ENREF_1)].

1. Wood BL, Arroz M, Barnett D, DiGiuseppe J, Greig B, et al. (2007) 2006 Bethesda International Consensus recommendations on the immunophenotypic analysis of hematolymphoid neoplasia by flow cytometry: optimal reagents and reporting for the flow cytometric diagnosis of hematopoietic neoplasia. Cytometry B Clin Cytom 72 Suppl 1: S14-22.

**Ferrell et al. – Supplementary Information S1 File**

|  | | **Supplemental Table B** – Patient characteristics and clinical outcomes | | | | | | | | | | | | | |
| --- | --- | --- | --- | --- | --- | --- | --- | --- | --- | --- | --- | --- | --- | --- | --- |
| **ID** | **Age** | | **Sex** | **Alive^§^** | **Induction Regimen^†^** | **Survival**  **(months)** | **BM**  **Blast** | **WBC** | **PB Blast** | **Cytogenetics** | **FLT3** | **NPM1** | **Induction 1** | **Induction 2** | **Relapse** |
| F001 | 23 | | F | + | IA | 14.5 | 97.5 | 432* | 95 | 46,XX,t(9;11)(p22;q23)[20] | Neg | Neg | CR | N/A | N/A |
| F002 | 77 | | M |  | Clof | 9 | 26 | 3.2 | 23 | 42~47,X,-Y,add(2)(p11.2), -5,add(6)(p12),add(7)(q22), add(12)(p13),del(12)(p12), -15,-18,-19,-20,+1~5mar | Neg | Neg | CR | N/A | 3 |
| F003 | 61 | | M |  | MA+Inv | 4.5 | 88.5 | 15.7 | 94 | 46,XY,del(11)(q21q23)[2]/46,XY[20] | Neg | Neg | Residual Leukemia | Residual Leukemia | N/A |
| F004 | 49 | | F | + | IA | 15 | 45 | 5.7 | 28 | 46, XX[20] | Neg | Neg | Residual Leukemia | CR | N/A |
| F007 | 59 | | F | + | DA | 14 | 52 | 1.8 | 0 | 47,XX,+22[3]/46,XX[17] | Neg | Neg | CR | N/A | N/A |
|  | | **^§^** ‘+’ indicates patient was alive at time of last follow-up.  * Patient F001 underwent leukapheresis prior to treatment and sample collection.  **^†^**  Regimens: IA – Idarubicin + cytarabine, Clof- Clofarabine, MA+Inv – Mitoxantrone + cytarabine + investigational agent, DA – Daunorubicin + cytarabine  WHO Classification available for F001 - AML with t(9;11)(p22;q23); MLLT3-MLL, F002 – AML with myelodysplasia related changes. For F003, F004, F007 there is no formal WHO classification available beyond AML, not otherwise specified. | | | | | | | | | | | | | |

**Ferrell et al. – Supplementary Information S1 File**


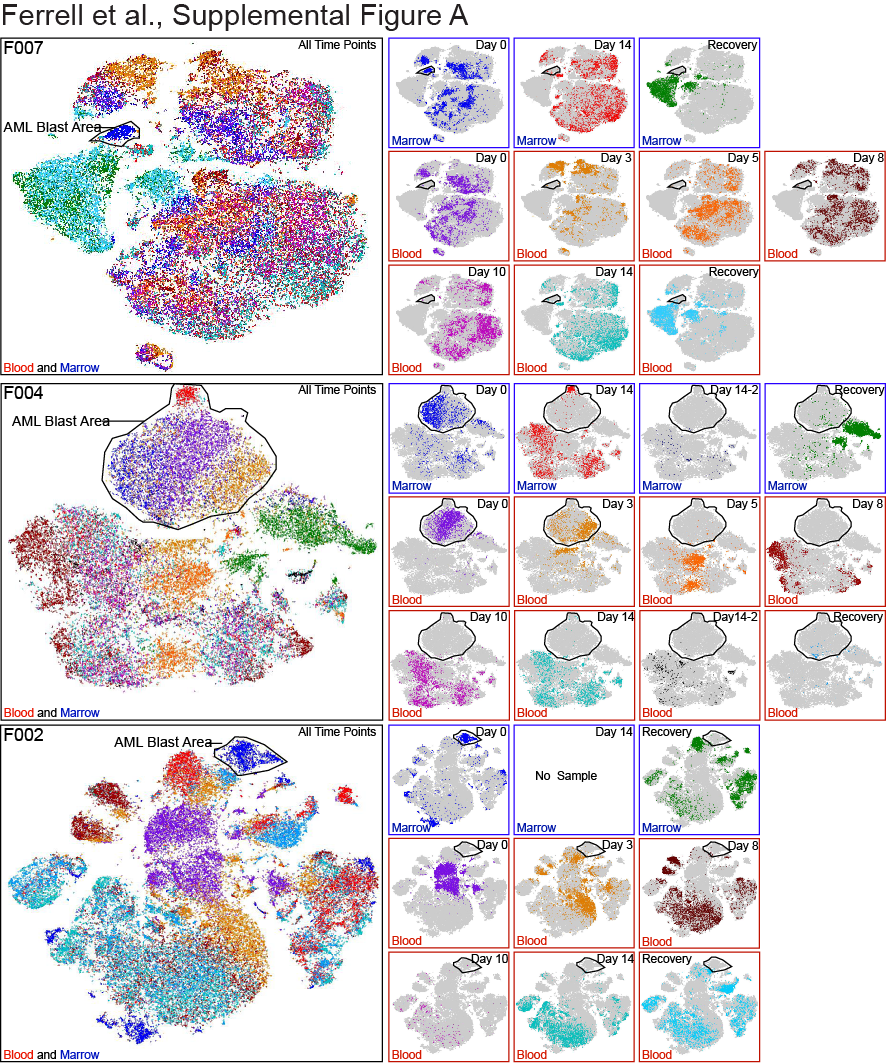


**Figure A – Comprehensive analysis of AML therapy response kinetics.** viSNE analysis characterizes changes in leukemia cell phenotype over time for cells from all treatment times for three individuals. All cells from all clinical time points were analyzed using viSNE according to the 27 markers measured (Supplemental Table S1). An AML blast area was identified as in Fig 1. Color indicates clinical time point and source, either bone marrow or blood. Patients F002 and F007 had very low blast percentages in the peripheral blood at diagnosis.

**Ferrell et al. – Supplementary Information S1 File**

**Supplemental Figure B**


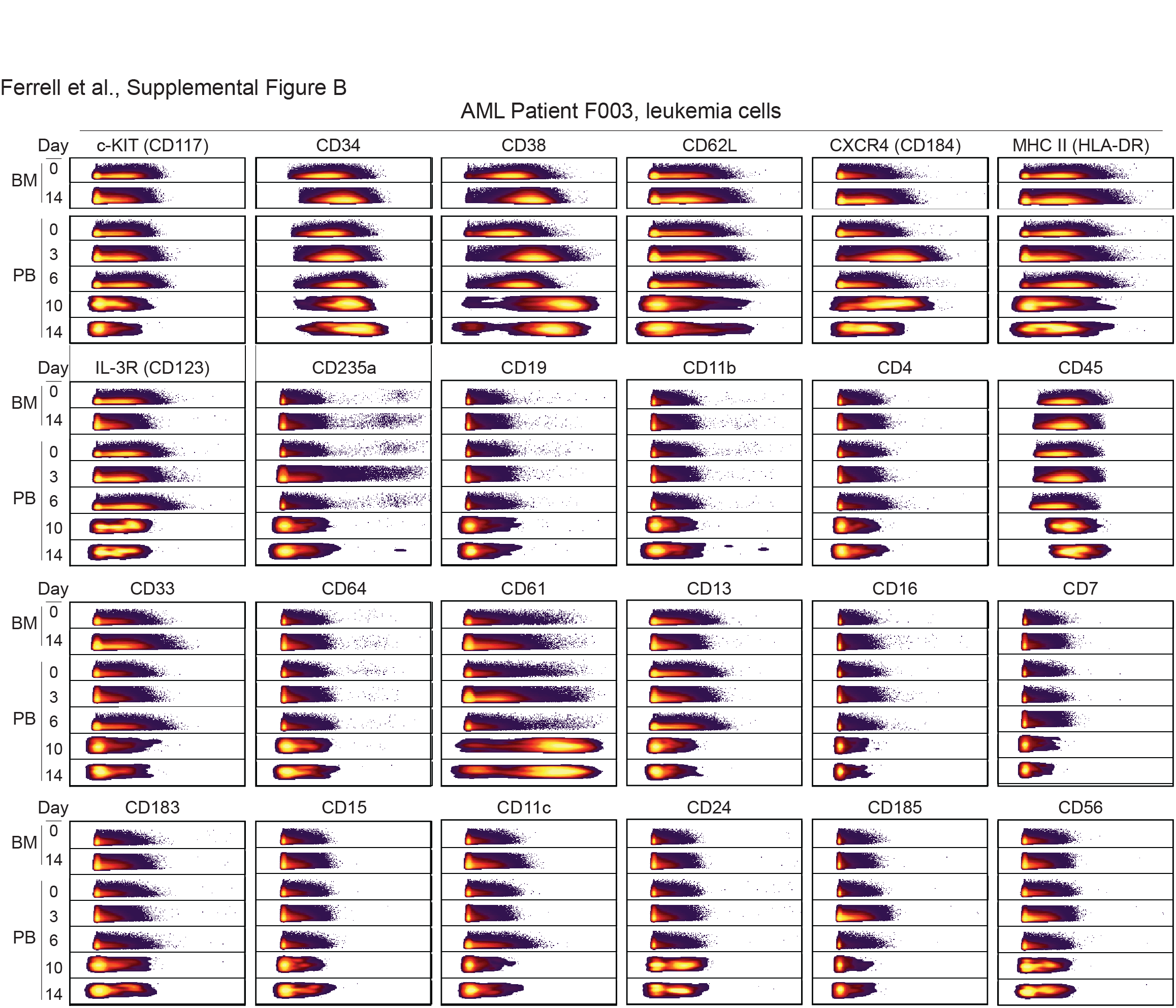


**Figure B - Changes in individual markers over time during treatment on AML blasts from patient F003.** Biaxial plots summarize six clinical time points (rows) for 24 markers (sets) for the AML blast cells from patient F003, gated as shown in Fig 3. The indicated marker is plotted on the x-axis using the same arcsinh_15_ scale as in other figures (e.g. Fig 1B). Plot labels are omitted to save space. The y-axis is mass cytometry event length, which is used here to spread the events out in the y-axis to create a compressed band plot view that allows rare subsets to be observed (see e.g. CD235a) that would be obscured in a traditional 1D histogram view.

**Supplemental Figure C**


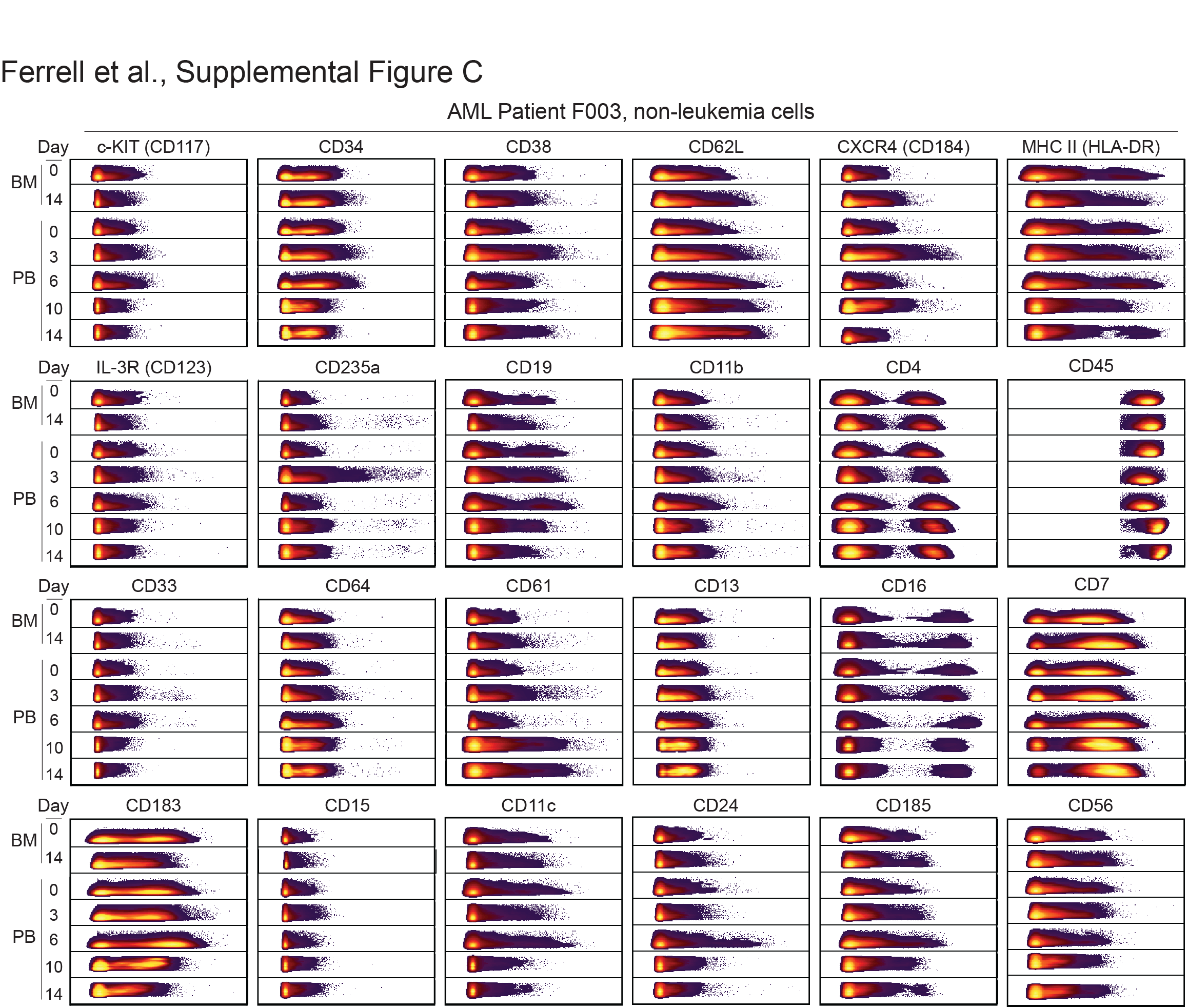


**Figure C - Changes in individual markers over time during treatment on non-leukemia cells from patient F003.** As in Fig B of S1 File, biaxial plots summarize six clinical time points (rows) for 24 markers (sets) for the non-leukemia cells from patient F003, gated as everything not in the leukemia blast gate shown in Fig 3. The indicated marker is plotted on the x-axis using the same arcsinh_15_ scale as in other figures (e.g. Fig 1B). Plot labels are omitted to save space. The y-axis is mass cytometry event length, which is used here to spread the events out in the y-axis to create a compressed band plot view that allows rare subsets to be observed (see e.g. CD16) that would be obscured in a traditional 1D histogram view.
